# Towards development of aptamers that specifically bind to lactate dehydrogenase of *Plasmodium falciparum* through epitopic targeting

**DOI:** 10.1186/s12936-018-2336-z

**Published:** 2018-05-03

**Authors:** Kelly-Anne Frith, Ronen Fogel, J. P. Dean Goldring, Robert G. E. Krause, Makobetsa Khati, Heinrich Hoppe, Mary E. Cromhout, Meesbah Jiwaji, Janice L. Limson

**Affiliations:** 1 Biotechnology Innovation Centre, Rhodes University, P.O. Box 94, Grahamstown, 6140 Eastern Cape South Africa; 2 Department of Biochemistry, Genetics and Microbiology, University of KwaZulu-Natal, Private Bag X01, Scottsville, 3209 KwaZulu-Natal South Africa; 3 Emerging Health Technologies Platform, Biosciences Division, Council for Scientific and Industrial Research, PO Box 395, Pretoria, 0001 Gauteng South Africa; 4 Department of Biochemistry and Microbiology, Rhodes University, P.O. Box 94, Grahamstown, 6140 Eastern Cape South Africa

**Keywords:** Aptamer, Oligonucleotide, Lactate dehydrogenase, Malaria, Biorecognition, Detection, SELEX

## Abstract

**Background:**

Early detection is crucial for the effective treatment of malaria, particularly in those cases infected with *Plasmodium falciparum.* There is a need for diagnostic devices with the capacity to distinguish *P. falciparum* from other strains of malaria. Here, aptamers generated against targeted species-specific epitopes of *P. falciparum* lactate dehydrogenase (r*Pf*LDH) are described.

**Results:**

Two classes of aptamers bearing high binding affinity and specificity for recombinant *P. falciparum* lactate dehydrogenase (r*Pf*LDH) and *P. falciparum*-specific lactate dehydrogenase epitopic oligopeptide (LDHp) were separately generated. Structurally-relevant moieties with particular consensus sequences (GGTAG and GGCG) were found in aptamers reported here and previously published, confirming their importance in recognition of the target, while novel moieties particular to this work (ATTAT and poly-A stretches) were identified. Aptamers with diagnostically-supportive functions were synthesized, prime examples of which are the aptamers designated as LDHp 1, LDHp 11 and rLDH 4 and rLDH 15 in work presented herein. Of the sampled aptamers raised against the recombinant protein, rLDH 4 showed the highest binding to the target r*Pf*LDH in the ELONA assay, with both rLDH 4 and rLDH 15 indicating an ability to discriminate between r*Pf*LDH and r*Pv*LDH. LDHp 11 was generated against a peptide selected as a unique *P. falciparum* LDH peptide. The aptamer, LDHp 11, like antibodies against the same peptide, only detected r*Pf*LDH and discriminated between r*Pf*LDH and r*Pv*LDH. This was supported by affinity binding experiments where only aptamers generated against a unique species-specific epitope showed an ability to preferentially bind to r*Pf*LDH relative to r*Pv*LDH rather than those generated against the whole recombinant protein. In addition, rLDH 4 and LDHp 11 demonstrated in situ binding to *P. falciparum* cells during confocal microscopy.

**Conclusions:**

The utilization and application of LDHp 11, an aptamer generated against a unique species-specific epitope of *P. falciparum* LDH indicated the ability to discriminate between recombinant *P. falciparum* and *Plasmodium vivax* LDH. This aptamer holds promise as a biorecognition element in malaria diagnostic devices for the detection, and differentiation, of *P. falciparum* and *P. vivax* malaria infections. This study paves the way to explore aptamer generation against targeted species-specific epitopes of other *Plasmodium* species.

## Background

In 2015, malaria was found to be endemic in 91 countries worldwide [1]. The World Health Organization (WHO) estimated that 212 million new cases of malaria were contracted in 2015, the majority being caused by the *Plasmodium falciparum* infections. An estimated 92% of the approximately 429,000 malaria-related fatalities occurred in Africa during 2015; the majority (≈ 70%) of these occurring in children under the age of five [1]. Being one of the leading causes of death in under-developed countries, early detection is crucial for the effective treatment of malaria, particularly in individuals infected with *P. falciparum*. There is a need for portable, simple, sensitive, reliable, accurate, durable, self-validating and cost-effective technologies for the rapid detection of malaria. Moreover, there is demand for a technique that can distinguish between the five separate species of parasites causing malaria in humans [2].

Although positive strides in the field of malaria diagnostics have been made in recent years, there still remain areas in which improvements can be made. Current methods of malaria diagnosis include: colourimetry [3]; fluorescence [4]; polymerase chain reaction (PCR; [5]); mass spectroscopy [6]; microfluidic cell enrichment [7]; magnetic resonance relaxometry [7]; loop-mediated isothermal amplification [8]; and, the gold standard: microscopy [4]. Although these techniques may be sensitive, they remain laboratory-confined and require skilled personnel and expensive equipment.

Immunochromatography is widely utilized in rapid diagnostic tests (RDTs) and makes use of antibodies as capture and detection bioagents in diagnosis. RDTs offer significant advantages over aforementioned techniques: rapid and accurate results; cost-effectiveness; ease of use and interpretation and portability, which allow these techniques to be used for in situ applications [9]. However, there is a need for diagnostic tests that have the capacity to distinguish between different species of malaria. For example, there have been reports that the pan-specific anti-malarial antibodies used in some RDTs have lowered affinities towards *Plasmodium ovale* and *Plasmodium malariae*, thus exhibiting lower detection sensitivities for these species [10]. *P. falciparum* is the most common malarial species infecting humans in sub-Saharan Africa and tests capable of accurately distinguishing it from other species are desirable.

*Plasmodium* lactate dehydrogenases (*P*LDH) are cytosolic homotetrameric enzymes that present a viable biomarker for RDT-based diagnosis. Not only is *P*LDH expressed throughout all stages of the *Plasmodium* spp. life cycle, but there is a high degree of sequence conservation within a species [11], as well as significant sequence dissimilarity to human analogues. Pan-specific epitopes on the surface of *P*LDH have been reported and validate the use of epitopes as biomarkers for the broad biorecognition of *Plasmodium* spp. [12]. Unique species-specific epitopes also on the surface of *P*LDH have been identified to discriminate between different species of malaria [12]. Similarly, peptides have been identified on the surface of GAPDH and shown to have the potential to differentiate between malaria species [13]. The synthesis of these unique epitopes for *P*LDH paves the way for the development of diagnostic techniques for in vitro and in vivo testing [14]. Hurdayal et al. [12] demonstrated that—by raising antibodies against synthesized oligopeptides corresponding to the species-specific epitopes (each approximately 13 amino acid residues long)—selective bioaffinity was observed between *P*LDH expressed by *P. falciparum* and *Plasmodium vivax*, amongst others. Therefore, by raising antibodies against the species-specific peptide sequences, the required increased level of bioaffinity specificity may be achieved [9, 12]. Many of the present commercial *Pf*LDH RDTs have variable results owing to their sensitivity towards extreme heat and humidity—which often decreases the efficacy of antibody-based techniques [8, 15, 16].

Aptamers are short, single-stranded biopolymers (nucleic acids, peptides, etc.) which offer high binding affinity and specificity to the target molecules that they are selected against. Aptamers represent alternative biorecognition agents to the more costly, but conventional, antibodies [17, 18]. Aptamers are generated from a vast library of candidate sequences in vitro using a technique called systemic evolution of ligands by exponential enrichment (SELEX) [17]. As biorecognition molecules, aptamers offer many advantages over antibodies, such as stability and robustness, ease of production, lowered sensitivity to heat and humidity, ease of modification, low immunogenicity and ability to be generated against a wide range of targets [19]. Previous ssDNA oligonucleotide aptamers generated against *P*LDH, used as a proteinaceous biomarker for malaria infection, did not exhibit species discrimination [20–23]. Strides towards the specific detection of *Pf*LDH over *Pv*LDH have been made recently by Cheung et al. [24] using aptamers generated against whole recombinant *Pf*LDH.

Given the higher prevalence, virulence and deleterious effects of *P. falciparum* infection over other plasmodial species, the work presented herein details the generation of aptamers with potential as biorecognition agents in biosensors capable of diagnosing malaria infection and, furthermore, distinguishing *P. falciparum* from other species of malaria. The approach taken to discriminate between *Plasmodium* spp. in this study was to generate single-stranded DNA/oligonucleotide aptamers against an oligopeptide corresponding to the *P*LDH epitope, LISDAELEAIFDC, unique to *P. falciparum*. This work reports on the generation of nine oligonucleotide sequences (aptamers) generated separately against the whole recombinant *Pf*LDH protein and a *P. falciparum*-specific lactate dehydrogenase (*Pf*LDH) epitopic oligopeptide (LDHp).

## Methods

### Recombinant protein and peptide synthesis

Recombinant proteins corresponding to lactate dehydrogenase enzymes from *P. falciparum* (r*Pf*LDH) and *P. vivax* (r*Pv*LDH) were expressed and purified as described in Hurdayal et al. [12]. The *P. falciparum*-specific LDH peptide epitope (LDHp) identified in Hurdayal et al. [12]—LISDAELEAIFDC—was synthesized by GL Biochem (China). All chemicals and reagents were purchased at Sigma Aldrich (Germany) unless specified otherwise.

### Isolation of aptamers against recombinant *Pf*LDH and LDH peptide

Aptamer synthesis was performed according to Rotherham et al. [25]. A library with single-stranded DNA (ssDNA) sequences (90 bases in length, with a 49 nucleotide length of randomized sequence flanked by constant regions for primer annealing) was sourced from Integrated DNA Technologies (IDT; USA). The library had the general sequence of 5′-GCCTGTTGTGAGCCTCCTAAC(N_49_)CATGCTTATTCTTGTCTCCC-3′.

Identification of oligonucleotides binding to *rPf*LDH and LDHp took place via parallel SELEX processes [17]. Selection using nitrocellulose membrane filtration at the initial phase of SELEX was adapted from previous protocols [26, 27].

Prior to use, nitrocellulose membranes (pore size of 0.45 µm, Merck Millipore, USA) were prepared by immersion in 0.5 M KOH for 20 min, rinsing with Milli-Q H_2_O, further incubation in 0.1 M Tris, pH 7.4 for 45 min and finally rinsing with HMCKN buffer (2 mM HEPES, 0.2 mM MgCl_2_, 0.2 mM CaCl_2_, 0.2 mM KCl and 15 mM NaCl, pH 7.4).

The ssDNA library was prepared in HMCKN to a final concentration of 1.59 μM. This was heat denatured at 95 °C for 10 min, cooled to − 20 °C for 5 min and equilibrated at room temperature for 5 min before being passed through prepared nitrocellulose membrane to remove non-specifically binding sequences (negative selection). The eluent was incubated with solutions of 1.59 μM target protein/peptide prepared in HMCKN; candidate aptamers were allowed to bind to the target at room temperature for 1 h under mild agitation. Following incubation, target-library mixtures were then passed through fresh nitrocellulose membranes (positive selection), which were then rinsed with HMCKN buffer to remove all unbound sequences. It was assumed that all protein/target-aptamer complexes were retained on the nitrocellulose filter [27].

Retained ssDNA-target complexes were eluted into 100 µl elution buffer (7 M urea, 100 mM citrate buffer and 3 mM EDTA, pH 8.0), by heating to 100 °C for 5 min. Eluted ssDNA was precipitated via phenol–chloroform extraction. 600 µl of phenol: chloroform:isoamyl alcohol mixture (25:24:1, saturated with 10 mM Tris, pH 8.0, 1 mM EDTA) was added to the elution buffer. This was mixed, incubated under agitation at room temperature for 30 min and centrifuged for 5 min at 7400×*g*. The aqueous phase was removed and set aside. To maximize ssDNA collection, an additional volume of 100 µl of sterile Milli-Q water was added to the organic phase; the suspension thoroughly remixed and centrifuged for 5 min at 7400×*g*. The aqueous phase was collected and combined with the previous aqueous phase. The combined aqueous phases were re-extracted using the same protocol described above.

The extracted ssDNA was precipitated in a manner similar to that described elsewhere [28] through the addition of 30 µl of 3 M sodium acetate buffer, pH 5.2, 3.3 µl of glycogen (20 g/l) and 1 ml of absolute ethanol. This was incubated for 16 h at − 80 °C and thereafter centrifuged at 4 °C for 30 min at 7400×*g*. The supernatant was carefully decanted and 1 ml of 80% (v/v) ethanol used to resuspend the white precipitate formed during centrifugation. The resultant mixture was centrifuged for a further 5 min at 7400×*g* at 4 °C. The ethanol was decanted and the pellet allowed to air dry at room temperature. The ssDNA pellet was resuspended in 30 µl sterile Milli-Q water.

The concentration and purity of ssDNA obtained during SELEX was quantified using a NanoDrop 2000 Spectrophotometer (ThermoScientific, USA). The concentration of extracted ssDNA was used to calculate the total mass of ssDNA binding to the target during the selection rounds (described as “ssDNA out”). Using the mass of ssDNA initially used in the selection (described as “ssDNA in”), the yield of positively binding ssDNA was calculated using Eq. 1:

1 $${\text{Recovery }}\left( \% \right) = \frac{{{\text{ssDNA out}} \left( {\text{ng}} \right)}}{{{\text{ssDNA}} {\text{in}} \left( {\text{ng}} \right)}} \times 100$$

Amplification of ssDNA was performed by PCR, producing amplified double-stranded DNA (dsDNA). PCR reaction mix (GoTaq^®^ Flexi DNA Polymerase kit, Promega, USA) prepared as per manufacturer’s instruction, and PCR generally proceeded using: 0.2 mM dNTPs (Fermentas, Thermo Scientific, USA); 3.5 mM MgCl_2_; 0.5 µM forward primer (5′-GCCTGTTGTGAGCCTCCTAAC-3′) (IDT, USA); 0.5 µM reverse primer (5′-GGGAGACAAGAATAAGCATG-3′) (IDT, USA); and, 10 µg/ml BSA (New England Biolabs, UK). The reverse primer was procured modified with an additional phosphate group at the 5′-end for lambda exonuclease digestion [27]. The temperature profile used for PCR was as follows: 95 °C for 3 min (initiation step); 4–20 cycles at 95 °C for 1 min (denaturation), 59 °C for 1 min (annealing) and 72 °C for 1.5 min (elongation); and, 72 °C for 8 min (final elongation step) on a MJ Mini Personal Thermo Cycler (Bio-RAD, USA). The MgCl_2_ concentration was decreased to 1.5 mM after the third round of SELEX to reduce the mutation rate.

The number of PCR cycles required optimization during every amplification stage of each round in SELEX to prevent over-amplification of the dsDNA and minimize the inclusion of amplification artefacts. This was achieved by performing a 200 µl pilot PCR run in each round, in which 20 µl reaction tubes were taken out after every second cycle following four rounds of PCR, e.g. 0, 4, 6, 8, 10, 12, 14, 16, 18, 20 cycles, with the blank control undergoing the maximum number of PCR cycles. Following identification of the optimum number of cycles, full-scale PCR with a reaction volume between 1.0 and 5.0 ml was performed.

PCR products were purified using the Nucleospin^®^ Gel and PCR clean-up kit, following the manufacturer’s instructions (Macherey–Nagel GmbH & Co. KG, Germany). Purified dsDNA was eluted in 50 µl sterile Milli-Q water. The quality of dsDNA was determined by PAGE using 8% (w/v) polyacrylamide gels electrophoresed at ≤ 120 V for 20–30 min in TBE buffer (45 mM Tris base, 45 mM boric acid, 1.3 mM EDTA, pH 8.0). Subsequently, gels were stained with 2.5 µM ethidium bromide solution or GelRed (Biotium, USA) and visualized under UV transillumination with a ChemiDoc XRS+ Molecular Imaging System (BioRAD, USA); the concentration of DNA was separately determined spectrophotometrically. To remove amplification artefacts occurring after Round 3, amplified DNA was purified by gel excision by initially electrophoresing the dsDNA on a 2.5% agarose gel at 80 V for 1.5–2 h in TBE buffer.

Following sufficient amplification of dsDNA (≥ 4.5 µg dsDNA), dsDNA was converted to ssDNA by lambda exonuclease digestion (New England Biolabs, UK), carried out for 4 h at 37 °C with a rate of ~ 1 U exonuclease per microgram dsDNA [28].

The ssDNA was purified using the Nucleospin^®^ Gel and PCR clean-up kit (Macherey–Nagel GmbH & Co. KG, Germany) as per manufacturer’s instruction. The resulting ssDNA was used for selection during the following round of SELEX. Eight rounds of selection were performed during SELEX.

The dsDNA from the final three rounds of SELEX was pooled, digested with lambda exonuclease to produce ssDNA and a final selection cycle was performed. Thereafter, the amplified dsDNA pool was ligated into the pGEM-T Easy vector (Promega, USA) and transformed into competent *Escherichia coli* JM109 cells (Rhodes University), according to the manufacturer’s instructions. Blue/white screening was conducted in which the cells containing ligated insert DNA (white colonies) were selected.

Sixteen (16) white colonies (containing oligonucleotide fragments selected against r*Pf*LDH) and 18 white colonies (containing oligonucleotide fragments selected against LDHp) were re-streaked onto a second set of Luria agar plates, and were hence selected for fragment length screening via PCR amplification. PCR-amplification took place using the pUC/M13 universal primers (IDT, USA) flanking the insert region of the pGEM-T Easy vector. Colonies containing the correct insert size—8 out of 16 for r*Pf*LDH and 8 out of 18 for LDHp—were thereafter resuspended in sterile Milli-Q water, heated to 95 °C for 10 min and PCR amplified using the KAPATaq kit (Kapa Biosystems, South Africa). The PCR reaction mix was prepared according to the manufacturer’s instructions and contained 0.1 mM dNTPs (Fermentas, Thermo Scientific, USA), 1.0 mM MgCl_2_, 0.2 μM 5′-biotinylated forward primer (5′-GCCTGTTGTGAGCCTCCTAAC-3′) (IDT, USA), 0.2 µM 5′-phosphorylated reverse primer (5′-GGGAGACAAGAATAAGCATG-3′) (IDT, USA) and 1 µl of resuspended cell debris. 30 cycles of PCR were performed to amplify the DNA, using the same temperature profile details for aptamer amplification. Amplified dsDNA was exonuclease-digested to ssDNA as previously described.

Biotinylated ssDNA sequences obtained in the above manner were used in a preliminary binding assay using ELONA, as detailed in the proceeding subsection. Colonies containing sequences that showed positive binding at the preliminary phase—4 out of 8 for r*Pf*LDH and 5 out of 8 for LDHp—were sequenced (Inqaba Biotec, South Africa) using the pUC/M13 universal forward and reverse primers. These sequences were selected for commercial synthesis (IDT, USA): rLDH 1, 4, 7 and 15; LDHp 1, 3, 11, 14 and 18 and pL1 [20, 22]; modifications for commercially-synthesized aptamers were 5′-biotin (for ELONA), and 5′-FITC (for confocal microscopy).

### Secondary structure prediction of aptamers

Preliminary identification and comparison of secondary structures of the aptamer sequences identified for both r*Pf*LDH- and LDHp-targeting sequences was performed using the Mfold server (http://mfold.rna.albany.edu/?q=mfold/dna-folding-form; [29]). To maintain parity with the SELEX conditions, structure prediction was conducted using the following environmental constraints: temperature was constrained to 23 °C; the sodium (Na^+^) and magnesium (Mg^2+^) concentrations were set to 15.0 mM and 0.2 mM, respectively.

### Enzyme-linked oligonucleotide assay (ELONA) of ssDNA aptamers

Binding of the selected synthesized oligonucleotides to their respective targets and control proteins (human serum albumin (HSA), mammalian (bovine) lactate dehydrogenase (mLDH) and recombinant *Plasmodium vivax* LDH (r*Pv*LDH)) was evaluated using the enzyme-linked oligonucleotide assay (ELONA) [30].

Briefly, 500 ng of protein, dissolved in 10 mM NaHCO_3_ buffer pH 8.5, was added to the wells of a 96-well ELISA plate and incubated overnight in a covered plate at 4 °C. Plates were washed three times with 300 µl of 25 mM Tris-buffered saline, pH 7.6, containing 0.1% (v/v) Nonidet P-40 (NP-40) (Roche, Germany), designated TBS+ hence forth. Wells were then blocked using 100 µl of 2% (w/v) fat-free milk solution in TBS+, incubating for 1 h at 4 °C. Plates were washed three times with 300 µl TBS+. Heat-activated biotinylated oligonucleotides were dissolved in HMCKN to the appropriate concentration (200 nM for the single-point analyses, and 50, 100, 250, 500 and 1000 nM for kinetic analyses), added to the allocated sample well, and incubated at room temperature for 2 h. Plates were then washed twice with 300 µl HMCKN buffer, followed by a further three washes with 300 µl TBS+. Streptavidin-linked horseradish peroxidase (SA-HRP; Kirkegaard and Perry Laboratories, USA) was diluted 1:1000 in TBS+; 100 µl of this solution was added to each well. Plates were covered and incubated at 37 °C for 2 h and unbound SA-HRP removed from the system by rinsing each well 4 times with 300 µl TBS+. Thereafter, 50 µl of the TMB solution consisting of the chromogenic substrate, TMB (3,3′,5,5′-tetramethylbenzidine), and hydrogen peroxide (Pierce, USA) was added to each well. Attached SA-HRP was monitored by the blue colour formed by the enzymatic oxidation of TMB in the presence of hydrogen peroxide [31]. After 8 min, 50 µl of 2 M sulphuric acid (stop solution) was added to each well to stop the enzymatic reaction producing a yellow colour. Absorbance values at 450 nm were then read for each well using the Powerwavex multiplate reader (Bio-Tek Instruments, USA). All measurements were performed in minimum triplicate (*n *= 3).

### Confocal microscopy

*Plasmodium falciparum* parasites (3D7 strain) were cultured in RPMI 1640 medium supplemented with 25 mM HEPES, 22 mM glucose, 0.65 mM hypoxanthine, 0.05 mg/ml gentamicin, 0.5% (w/v) Albumax II and 3% (v/v) human red blood cells. Cultures were maintained at 37 °C in sealed culture flasks suffused with a 5% CO_2_, 5% O_2_, 90% N_2_ gas mixture. When the culture contained predominantly mature stage parasites (trophozoites and schizonts) as judged by light microscopy of Giemsa-stained blood smears, the red blood cells were pelleted, washed and resuspended in PBS. Round glass coverslips (12 mm diam.) were coated for 15 min with 1 mg/ml poly-l-lysine at room temperature. The glass coverslips were rinsed with 1 ml of 1× PBS, pH 7.4. *P. falciparum*-infected red blood cells, suspended in PBS were allowed to settle on the poly-l-lysine coated coverslips for 1 h. Unbound red blood cells were gently washed away using PBS. Bound red blood cells were lysed with 0.05% saponin for 1 min and rinsed with PBS to remove haemoglobin and the remaining cell debris from the immobilized *P. falciparum* parasite bodies. Parasite bodies were fixed to the glass coverslips using 1 min incubation with ice-cold methanol. Unfixed parasite bodies were removed with a PBS wash. The coverslips were then blocked with 100 mg/ml HSA in PBS for 20 min.

As a positive control, samples were also incubated for 45 min with IgY generated to the species-specific *P. falciparum* epitope (described by Hurdayal et al. [12]), followed by three washes with PBS, pH 7.4. To elicit a fluorescent response, the antibody control included a 45 min incubation with fluorescein-tagged donkey anti-chicken IgG (Biotium, Inc., USA) as secondary antibody. The fixed parasites were incubated in the dark with 200 nM heat-activated 5′-modified FITC-tagged aptamer in HMCKN buffer (as previously described) for 45 min. Coverslips were washed three times with PBS, pH 7.4. Fixed parasite bodies were incubated in 1 µg/ml DAPI in PBS for 1 min. Coverslips were briefly dipped in Milli-Q H_2_O, dried, mounted with Fluoroshield^®^ (ImmunoBioScience Corp., USA) and allowed to dry in the dark overnight.

Cells were imaged using a Zeiss LSM780 laser scanning confocal microscope (Carl Zeiss Microscopy GmbH, Jena, Germany) using the x63 objective. All images were acquired using the same exposure and detector settings for each spectral channel. The Zen 2011 Blue software was used to acquire images from the Zeiss LSM780 microscope and to perform image overlays. ImageJ 1.50i software was used for image analysis whereby areas of interest were highlighted and the integrated density value (IntDen) and area measured. GraphPad Prism 5 was used to plot the mean fluorescent intensity (IntDen/Area) values for 12 parasites of interest across three micrograph frames.

### Statistics

All measurements were performed in, at minimum, triplicate. Presented results are the means of measurements, while all reported error bars and uncertainties represent one standard deviation from the mean. Significant difference was identified using the Kruskal–Wallis *H* test and datasets significantly different from their counterparts identified using Dunn’s multiple comparison test statistical significance determined using a significance level, α, set to 0.05.

The apparent dissociation constants (K_D_) of aptamer-target interactions were calculated using the kinetic information obtained from ELONA analysis. ELONA assay responses were fitted via nonlinear regression (Least-Squares minimization) to a variant of a previously-described Langmuir equivalent binding isotherm equilibrium formula [32], using Statistica^®^. The formula is represented in Eq. 2:

2 $${\text{Assay response}}\; \left( {\Delta {\text{OD}}_{{450\;{\text{nm}}}} } \right) = \left( {\frac{{\varGamma_{\text{max} } \times [{\text{aptamer}}]}}{{{\text{K}}_{\text{D}} + \left[ {\text{aptamer}} \right]}}} \right)$$

where [aptamer] is the concentration of the aptamer used during ELONA (M), ΔOD_450 nm_ is the change in the ELONA absorbance at a given [aptamer], relative to the assay response when [aptamer] = 0 M. These were used to calculate the K_D_ i.e. the apparent dissociation constant of the aptamer-target complex (M), and Γ_max_, the maximal assay response for the aptamer-target complex.

Averages and standard errors of K_D_ and Γ_max_ are presented. In addition to presenting these values, a Wald test of the parameters was included to calculate the significance of the K_D_ and Γ_max_ nonlinear regression coefficients: *p* values less than 0.05 indicate values that the model assessed to be integral to the dependence of ΔOD_450 nm_ on [aptamer] for a given aptamer-target complex.

## Results

### Isolation of aptamers against recombinant *Pf*LDH and a *Pf*LDH-specific peptide

**Fig. 1:**
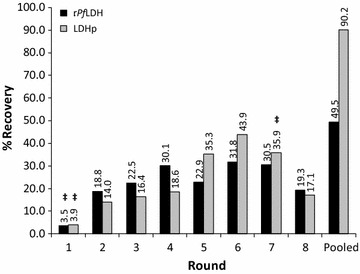
Percent (%) recovery of ssDNA during the SELEX process for the generation of aptamers from recombinant *Plasmodium falciparum* lactate dehydrogenase (r*Pf*LDH) and *Plasmodium falciparum*-specific peptide (LDHp) targets. ^‡^Denotes a negative selection step, in which ssDNA was passed through prepared nitrocellulose membranes lacking target protein, to remove non-specifically binding sequences

A modified exonuclease-based SELEX was performed from a library containing 10^14^–10^15^ oligonucleotides with 49 randomized nucleotides flanked by the constant regions. Eight rounds of SELEX were performed prior to combining dsDNA from rounds 6 through to 8, dubbing the final selection as “pooled” in order to enrich the sequence diversity. Successive enrichment, culminating in binding recoveries of 49.5 and 90.2% was observed for aptamers binding to the recombinant *P. falciparum* LDH protein (r*Pf*LDH) and *Pf*LDH-specific lactate dehydrogenase epitopic oligopeptide (LDHp), respectively (Fig. 1). The final dsDNA pool was then further amplified and ligated into the pGEM-T Easy vector as outlined in the “Methods” section.

Following insertion into plasmid vectors, blue-white screening utilizing disruption of the *β*-galactosidase gene positively identified clones containing the correctly-sized insert corresponding to introduction of aptamers, were then picked at random to undergo preliminary screening. Through PCR and subsequent exonuclease digestion, separate sequences modified at the 5′ site with biotin were obtained; these were used for preliminary ELONA. Clones that showed greater binding to their respective targets, r*Pf*LDH and LDHp, over their counterparts and control protein (data not shown) included rLDH 1, 4, 7 and 15 and LDHp 1, 3, 11, 14 and 18. These sequences were subsequently sequenced and synthesized with 5′-biotinylation for binding analysis reported in subsequent studies.

### Recombinant *Pf*LDH and *Pf*LDH peptide aptamer sequence analyses

A two-sequence homology alignment of synthesized oligonucleotides, using DNAMAN sequence analysis software (Lynnon Corporation, Canada), revealed a sequence homology between 25.5 and 47.1%, with an overall homology of 39.4% using a multiple sequence alignment. Generated sequences were then compared with those in literature. Percent homologies ranged from 30.6% for pL1 and rLDH 15–65.4% for pL2 and rLDH 1 [20]. When the synthesized aptamers were compared with those patented by Tanner et al. [21]. percent homologies ranged from 18.3% (2021s aligned with rLDH 15) to 38.8% (2009s aligned with LDHp 14).

The sequences of the variable region of the rLDH 1, 4, 7 and 15 and LDHp 1, 3, 11, 14 and 18 aptamers, the concatemer control, C7, as well as sequences of published *Plasmodium* LDH aptamers are listed in Table 1. Interestingly, a poly-A stretch in the sequences (namely rLDH 1, 4 and 15 and LDHp 14) is not uncommon. Reports show that these poly-A stretches tend to stack and form complex helical conformations [32–34] that are not immediately evident in secondary structure-predicting software, as was used for this study. They, therefore, are of interest and may play a role in the formation of the tertiary structure of these aptamers.

**Table 1:**
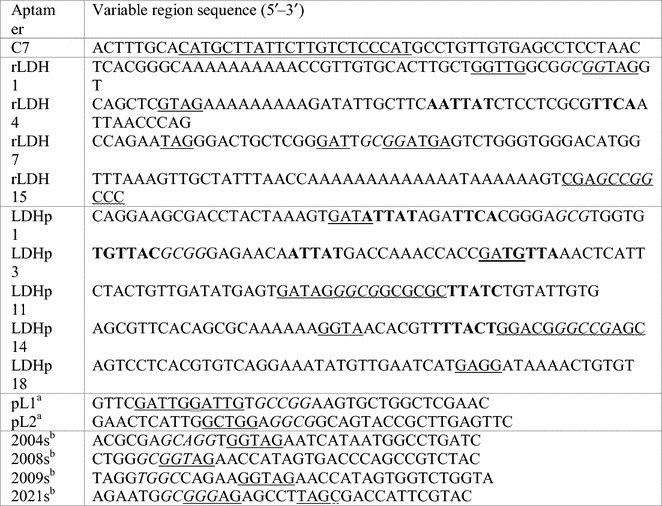
Sequence of the variable region for the ssDNA LDH aptamers generated with common moieties noted

Solid underlining: GGTAG-type moiety; bold: ATTAT-type moiety; italics: GGCG-type moiety; zigzagged underlining: GC-rich region

^a^r*Pf*/r*Pv*LDH aptamers from Lee et al. [20]

^b^r*Pf*LDH aptamers from Tanner et al. [21]

**Fig. 2:**
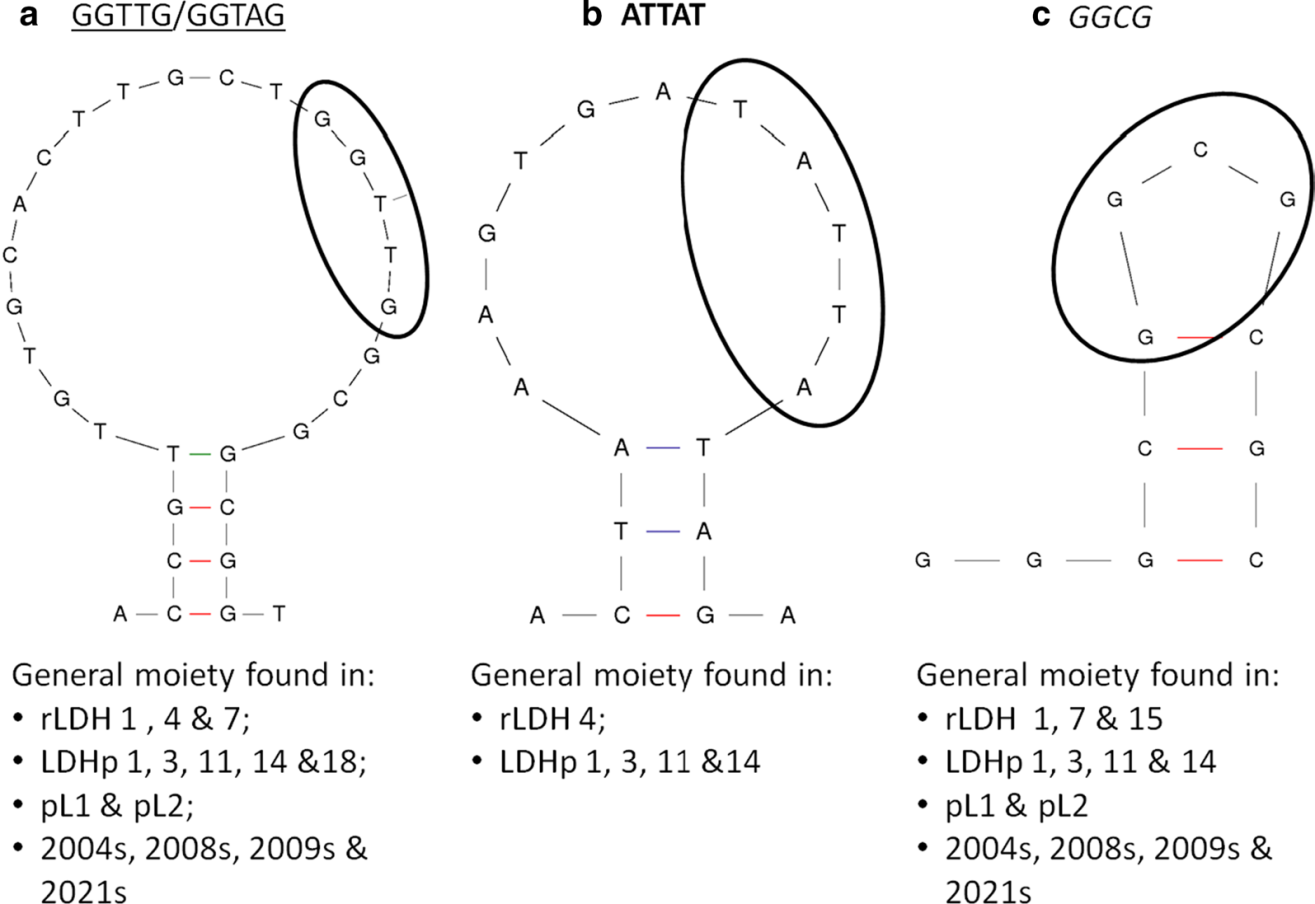
Moieties found on secondary structures of aptamers generated with examples of moiety location encircled (**a** *GGTTG/GGTAG; **b** ATTAT; **c** GGCG). *Moieties with the general sequence, GGTAG or GGTTG (differing by one nucleotide) are present on the loop structure of selected aptamers. The example shown here indicates GGTTG

The binding properties dictated by the primary and tertiary structures of protein-binding nucleic acid aptamers determine the location and affinity of the binding interaction [18]. Identification of sequence motifs and structural moieties assist in the understanding and determination of the protein target and aptamer interaction. Secondary structures (generated using the MFold software) were compared and conserved moieties in sequenced aptamers were identified. These areas of homology are highlighted and compared in Table 1 with one another, as well as to those generated by Lee et al. [20] and Tanner et al. [21]; and, locations compared with the secondary structures are presented in Fig. 2.

The moiety, ATTAT (Fig. 2b and in bold in Table 1), can also be found on the stem-loop structure of the synthesized aptamers. However, this particular moiety appears to occur frequently in the aptamers selected against the *P. falciparum*-specific peptide indicating its relevance in the tertiary structure of aptamers against the smaller LDHp target.

The sequence, GCGG and reverse of (GGCG) (in italics), is present in 10 of the 15 analysed sequences in Table 1. This sequence is present in the stem and stem-loop secondary structures of pL1, 2008s and LDHp 11 (Fig. 2c) and, thus too, forms an integral part of the structure necessary for recognition and binding of the aptamer to the r*Pf*LDH target. LDHp 11, LDHp 14 and rLDH 15 contain a GC-rich stem-loop structure (underlined with zig-zagging in Table 1) incorporating the GCGG moiety (shown in Fig. 2c).

### Binding analyses of recombinant *Pf*LDH and *Pf*LDH peptide aptamers

#### Enzyme-linked oligonucleotide assay (ELONA)

**Fig. 3:**
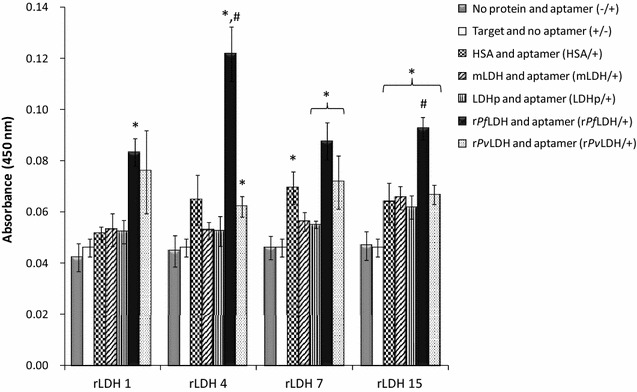
ELONA-assessed binding of biotinylated rLDH-generated ssDNA aptamers to the target, r*Pf*LDH, the polypeptide, LDHp, and relevant proteins used as controls in this study. *Indicates statistically-significantly larger ELONA responses compared to the absence of aptamers (*n* = 3; *p* ≤ 0.05); ^#^indicates statistically-significantly larger ELONA responses of the aptamers binding to the target protein, compared to the other proteins investigated (*n* = 3; *p* ≤ 0.05)

**Fig. 4:**
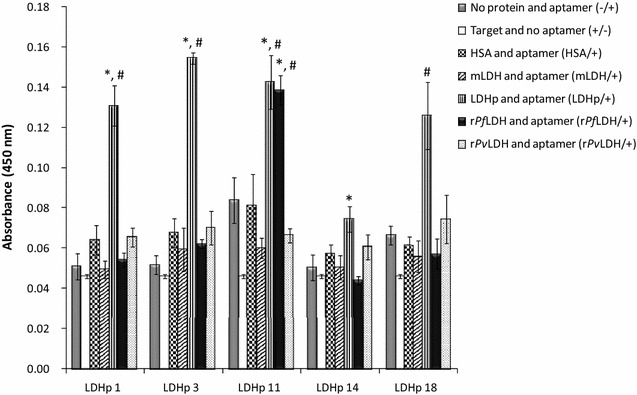
ELONA-assessed binding of biotinylated LDHp-generated ssDNA aptamers to the target, LDHp, whole r*Pf*LDH, and the relevant control proteins. *Indicates statistically-significantly larger ELONA responses compared to the absence of aptamers (*n* = 3; *p* ≤ 0.05); ^#^indicates statistically-significantly larger ELONA responses of the aptamers binding to the target protein, compared to the other proteins investigated (*n* = 3; *p* ≤ 0.05)

The extent and specificity of binding of each of the synthesized 5′-biotinylated aptamers to r*Pf*LDH (Fig. 3) and LDHp (Fig. 4) were performed using ELONA. Assay responses for each aptamer, given by the colourimetric optical density (OD) values at 450 nm, are indicative of an aptamer’s binding affinity to the immobilized target. Control proteins were also included in the analyses and included human serum albumin (HSA), mammalian LDH (mLDH)—both expected to be found in high concentrations in the analyte matrix—and recombinant *P. vivax* LDH (r*Pv*LDH), used to assess inter-species binding specificity. Single-point assay responses were used to screen the aptamers to multiple proteins, r*Pf*LDH, LDHp, r*Pv*LDH, HSA and mLDH, as similarly shown by Stoltenburg et al. [35] and Sypabekova et al. [36]. Of the sampled aptamers raised against the recombinant protein, rLDH 4 showed the highest binding to the target r*Pf*LDH in the ELONA assay.

As illustrated in Fig. 3, binding of the rLDH 4 aptamer to r*Pf*LDH was significantly higher (0.120 ± 0.011 OD) than binding of rLDH 1, 7 and 15 aptamers to r*Pf*LDH (0.083 ± 0.006 OD, 0.088 ± 0.005 OD and 0.093 ± 0.012 OD, respectively). Furthermore, binding of rLDH 4 aptamer to r*Pf*LDH produced a significantly higher colourimetric response than the same sequence binding to the control proteins mLDH (0.053 ± 0.003 OD), HSA (0.065 ± 0.010 OD) and r*Pv*LDH (0.062 ± 0.004 OD, significant vs. negative control). As indicated here the rLDH 4 aptamer exhibits a preference for r*Pf*LDH and discriminates between r*Pf*LDH and r*Pv*LDH, given that the signal against r*Pv*LDH does not differ from the HSA control.

Colourimetric assessment of rLDH 15 aptamer binding to the target r*Pf*LDH showed statistically-significantly higher binding compared to its binding to the control mLDH, HSA and r*Pv*LDH (Fig. 3) indicating that it exhibits species specificity. The ELONA study indicated lower binding to the target r*Pf*LDH by rLDH 15 compared to rLDH 4 (Fig. 3). Although rLDH 1 and rLDH 7 aptamers do show positive binding to r*Pf*LDH the high signal obtained for rLDH 1 binding to r*Pv*LDH suggests binding to a common motif shared between the proteins, while the high levels of rLDH 7 binding to HSA compared to r*Pf*LDH suggests that the aptamer may lack specificity for r*Pf*LDH in human serum samples.

None of the rLDH aptamers tested demonstrated statistically significant binding to LDHp (Fig. 3), either compared to control proteins or baseline assay responses. Given the low degree of consensus between the LDHp group of aptamers and the rLDH aptamers, this may indicate (Table 1) that the rLDH aptamers generated in this study bind to other sites than that modelled by LDHp.

Binding analysis of the pool of aptamers generated against the LDH epitopic oligopeptide indicated that four aptamers (LDHp 1, 3, 11 and 18) bind strongly to the target LDHp indicated by a higher absorbance at 450 nm (Fig. 4). These four LDHp aptamers had similar signal intensities with LDHp in the assay (with an average OD of 0.138 ± 0.013 OD for these four aptamers). The four aptamers demonstrated significant (*p* ≤ 0.05) binding to the target LDHp compared to mLDH, HSA, r*Pv*LDH and the other controls. Binding of these aptamers to the LDHp with similar OD values and the lack of binding to r*Pv*LDH, indicates discrimination of the aptamers between the selected *Plasmodium* spp. Only one of the four aptamers, LDHp 11, bound both the recombinant protein r*Pf*LDH and the peptide. The LDHp 11 aptamer exhibits selective recognition of the epitope when in the appropriate conformation in the recombinant protein (Fig. 4). This species-specificity facilitates the further development of a sensing technique by which to specifically detect *P. falciparum* in a biosensor.

#### Kinetics of binding affinity of tested aptamers for r*Pf*LDH and r*Pv*LDH using ELONA

**Fig. 5:**
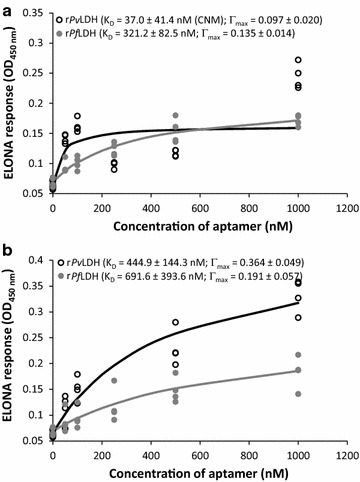
ELONA-assessed (A_450 nm_) binding kinetics of biotinylated **a** LDHp 11 and **b** rLDH 4 to r*Pf*LDH and r*Pv*LDH

The kinetics of the dependence of the aptamer concentration on the ELONA assay response was examined for aptamers identified in this study as capable of specific binding to the recombinant r*Pf*LDH protein, and examples of pan-specific pLDH aptamers reported in literature. To assess species-specificity of the aptamers to their LDH targets, binding kinetics for both *rPf*LDH and *rPv*LDH were assessed for each tested aptamer. Examples of binding kinetics and regression model fits for LDHp 11 and rLDH 4 to r*Pf*LDH and r*Pv*LDH determined using ELONA are shown in Fig. 5.

Table 2 shows the apparent affinity constants (K_D_) of generated aptamers, LDHp 1, LDHp 11, rLDH 4, rLDH 7, rLDH 15, and the C7 concatemer, as well as the previously published aptamers, pL1 [20, 22] and 2008s [21, 23, 24], to r*Pf*LDH and r*Pv*LDH, as determined through ELONA [32].

**Table 2:** K_D_ values for aptamers LDHp 1, LDHp 11, rLDH 4, rLDH 7, rLDH 15, C7 and pL1 binding to immobilized proteins, r*Pf*LDH and r*Pv*LDH determined using ELONA

Aptamer	Apparent affinity constant, K_D_ (nM) ± SE				References
	r*Pf*LDH		r*Pv*LDH		
	K_D_	Γ_max_	K_D_	Γ_max_	
C7	NR	NR	NR	NR	This work
LDHp 1	927.3 ± 915.0	0.068 ± 0.039	CNM > 1000	0.230 ± 0.106	This work
LDHp 11	321.2 ± 82.5*	0.135 ± 0.014*	37.0 ± 41.4 CNM	0.097 ± 0.020*	This work
rLDH 4	691.6 ± 393.6	0.191 ± 0.057*	444. 9 ± 144.3*	0.364 ± 0.049*	This work
rLDH 7	39.9 ± 15.7*	0.180 ± 0.014*	26.3 ± 3.2*	0.283 ± 0.050*	This work
rLDH 15	80.7 ± 17.1*	0.129 ± 0.007*	268.7 ± 67.2*	0.501 ± 0.429*	This work
pL1	159.5 ± 167.8 CNM	0.022 ± 0.005*	79.2 ± 12.7*	0.209 ± 0.010*	This work
	38.7 ± 1.3	–	16.8 ± 0.6	–	[20]
	6.2	–	2.9	–	[24]
2008s	42.0–59.0	–	–	–	[23]
	43.0	–	NR	–	[24]

*K*_*D*_ estimated apparent dissociation constant (M) of the aptamer-target complex, *Γ*_*max*_ estimated maximal assay response for the aptamer-target complex, *CNM* could not model—positive binding occurred, but no valid modelled K_D_ was obtained, *CNM > 1000* could not model—linear dependence indicates that apparent K_D_ of a target did not fall within the tested concentration range i.e. K_D_ > 1000 nM, *NR* no response—no evidence of binding, relative to the baseline assay response

* Wald test produced a probability, *p* of < 0.05 for this parameter

No detectable response (NR) was recorded for those aptamers that did not exhibit binding to the immobilized protein. Could not model (CNM) signifies aptamers for which maximal OD_450 nm_ responses greater than baseline assay responses were recorded i.e. Γ_max_ > 0, but in which K_D_ values could not be determined due to failure of the model e.g. a K_D_ with standard errors greater than their averages A designation of CNM > 1000 was made where linear dependence indicated that the apparent K_D_ of a target did not fall within the tested concentration range i.e. K_D_ > 1000 nM. The Wald test was performed to measure the validity of the measured parameter to the aptamer concentration-dependent OD_540 nm_ response: The Wald test assesses whether the parameter distribution within the confidence interval is necessary for the described mathematical dependence i.e. the certainty that K_D_ and Γ_max_ are > 0 for the dataset investigated. Failure of the Wald test for a given parameter tested using the model shown in Eq. 2 is indicated by *p* < 0.05.

Γ_max_ is an estimated parameter extrapolated from the degree of assay response recorded for aptamer-target kinetic ELONAs i.e. the ΔOD_450 nm_. As this inherently varied between kinetic studies due to slight differences in execution of the assay e.g. TMB substrate exposure time, it is impossible to compare this between different aptamer-target pairs to evaluate differences in binding affinity for this study. Hence, comparison of the kinetics is confined to the apparent dissociation constant, K_D_. Γ_max_, and the associated Wald test on that parameter, which was used to determine if statistically-significant binding between an aptamer and the protein target in a given study took place.

No detectable response was recorded for the negative concatemerized aptamer control, C7 (Table 2), indicating that the primer-binding sequences flanking the randomized region enriched during SELEX in this study did not, by themselves, exhibit binding to either r*Pv*LDH nor r*Pf*LDH, as expected of this negative control.

K_D_ values could not be determined for the binding of r*Pv*LDH to aptamers LDHp 11 and LDHp 1. For these aptamer-target complexes, the binding kinetics followed a linear, rather than hyperbolic trend within the concentration range used in this study. Linear dependence indicated that the apparent K_D_ of a target did not fall within the tested concentration range i.e. K_D_ > 1000 nM and resulted in a could not model (CNM > 1000) annotation for these aptamer-target pairs (Table 2).

Of the two peptide aptamers, LDHp 1 and LDHp 11, examined here, the modelled K_D_ between aptamer LDHp 11 and r*Pf*LDH of 321.2 ± 82.5 nM (Table 2) indicated preferential binding of this aptamer to r*Pf*LDH over r*Pv*LDH, evident in Fig. 4. The lack of Wald test significance and high variability in modelled K_D_ (927.3 ± 915.0 nM) and Γ_max_ (0.068 ± 0.039) for the binding of r*Pf*LDH to LDHp 1 largely indicates that this aptamer exhibited binding responses that were independent of aptamer concentration.

An examination of the aptamers generated against the whole recombinant protein, aptamer rLDH 4 exhibited higher modelled *K*_*D*_ values for r*Pf*LDH (691.6 ± 393.6 nM) compared to LDHp 11, indicating a lower affinity between this aptamer and the protein. The lower K_D_ of 444.9 ± 144.3 nM obtained for rLDH 4 and *rPvLDH* indicated a lack of species-specificity by this aptamer, albeit with a statistically insignificant difference (*p* > 0.05) between the K_D_ values for r*Pf*LDH and r*Pv*LDH for this aptamer. LDHp 11, thus, exhibits greater sensitivity and species-specificity, compared to rLDH 4, as not only is the K_D_ value of LDHp 11 for r*Pf*LDH lower, but LDHp 11 did not display valid modellable and measurable binding to r*Pv*LDH. Furthermore, and as shown in Fig. 4, aptamer LDHp 11 displayed greater binding to r*Pf*LDH over r*Pv*LDH given by the OD_450 nm_ responses for LDHp 11 binding to r*Pf*LDH being higher than that of r*Pv*LDH using static ELONA (Fig. 4).

A K_D_ of 80.7 ± 17.1 nM was determined for binding interactions between rLDH 15 and r*Pf*LDH showing stronger affinity for r*Pf*LDH than LDHp11. However, a K_D_ of 268.7 ± 67.2 nM between rLDH 15 and r*Pv*LDH shows preferential, but not specific, binding to r*Pf*LDH by this aptamer. Of the aptamers generated in this study, rLDH 7 exhibited the lowest K_D_ for both tested targets (39.9 ± 15.7 nM for r*Pf*LDH and 26.3 ± 3.2 nM for r*Pv*LDH, respectively), indicating similar high affinities to both targets. (rLDH 7 however also showed non-specific binding to HSA during ELONA studies). The values obtained here are nevertheless similar to those previously reported with a K_D_ of 42.0 (isothermal titration colorimetry [23]), 43.0 nM (ELONA [24]), 56 ± 18 nM (electromobility shift assay [23]) and 59 nM (surface plasmon resonance [23]) for aptamer 2008s generated against r*Pf*LDH (Table 2). Aptamer rLDH 15 will be investigated further for species-specific binding between r*Pf*LDH and r*Pv*LDH.

Previously published aptamer, pL1 [20], exhibited calculated K_D_ values of 79.2 ± 12.7 nM for r*Pv*LDH, one of the highest affinities recorded in this study for that protein. However, using the methods detailed in this study, this aptamer produced no valid modellable K_D_ for r*Pf*LDH, despite producing ELONA responses above the baseline, resulting in a CNM determination (Table 2).

#### In situ binding analysis using confocal microscopy

**Fig. 6:**
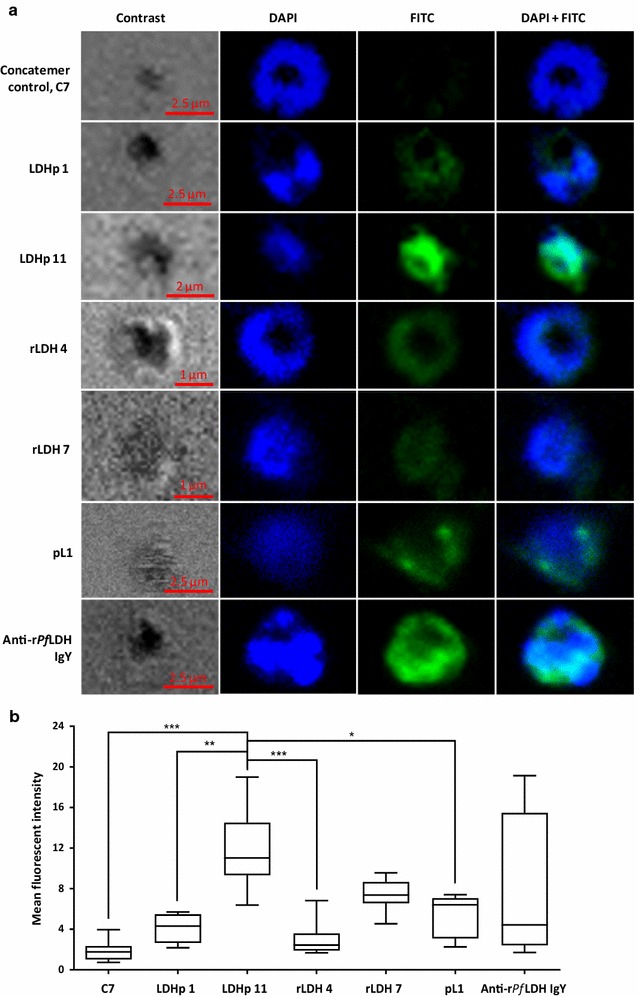
**a** Confocal micrographs of immobilized *P. falciparum* infected red blood cells stained with DAPI and either FITC-tagged aptamers or r*Pf*LDH-specific antibodies. **b** Quantification of FITC-tagged aptamers and r*Pf*LDH-specific antibody fluorescence associated with immobilized *P. falciparum* infected red blood cells (*n* = 12; *p* ≤ 0.05). FITC tagged aptamers included LDHp 1, LDHp 11, rLDH 4, rLDH 7, pL1 and concatemer, C7. IgY antibodies against r*Pf*LDH were detected with FITC-labeled secondary antibody. Left to right: phase-contrast; green channel (FITC; excitation = 490; emission = 525 nm); blue channel (DAPI; excitation = 360 nm; emission = 460 nm), and merged blue- and green-channel images. Each scale bar represents 2 µm. *Significant: *p* = 0.01–0.05 (Dunn’s multiple comparison test). **Very significant: *p* = 0.001–0.01 (Dunn’s multiple comparison test). ***Extremely significant: p < 0.001 (Dunn’s multiple comparison test)

In all instances of the preliminary confocal microscopy imaging (Fig. 6), DAPI was seen to bind strongly to the parasite’s nuclear material and, to a lesser extent, the outer membrane. The cytosolic localization of LDH is confirmed by the cellular fluorescence profile of anti-r*Pf*LDH IgY binding, in which the separation between the FITC-stained cytosol and nuclear material may be inferred.

The generated FITC-tagged aptamers, LDHp 1, LDHp 11, rLDH 4 and rLDH 7, appeared to bind to native LDH found in *P. falciparum* in blood cultures with a similar cytosolic profile to that obtained with the IgY antibody with comparable mean fluorescent intensities, compared to the low fluorescence exhibited by the negative control, concatemer C7. Aptamer LDHp 11, which demonstrated high binding affinity to the recombinant *Pf*LDH during ELONA studies (Fig. 4 and Table 2), exhibited a higher and statistically significant mean fluorescent intensity towards native *Pf*LDH compared to the concatemer control, C7, LDHp 1, rLDH 4 and pL1 (Fig. 6b). The control aptamer, pL1, also showed positive binding to native *Pf*LDH.

## Discussion

Aptamers are promising reagents for the detection of biological molecules, offering different characteristics to more traditional antibody based reagents. This potential has been explored for the detection of malaria lactate dehydrogenase [20–24] an established target for the diagnosis of malaria in antibody-based rapid diagnostic tests [9]. Aptamers with different structures to those reported previously, which can differentiate between malaria species, have been developed and are presented herein. Aptamers with promising binding characteristics were selected and included 4 aptamers against rLDH and 5 against the LDHp peptide. Aptamer sequences were compared with published sequences and shared 30–66% homology [20]. There was 18–39% homology with respect to patented sequences [21]. Interestingly, a poly-A stretch in the sequences rLDH 1, 4, 15 and LDHp 14 is common. These poly-A stretches tend to stack and form complex helical conformations that are not immediately evident in secondary structure-predicting software like that employed in this study [32–34]. The sequences may play a role in the formation of the tertiary structure of these aptamers. A GGTTG sequence common to eight aptamers selected has been described before and is thought to play a pivotal role in the recognition and binding of aptamers to *Plasmodium* LDH [23, 24]. The fact that the GGTTG has been identified in independent studies on malarial LDH, strongly support its importance in the binding of aptamers to LDH. This implies that the GGTTG structure binds to similar secondary structures on the surface of r*Pf*LDH and may be dominant docking sites for aptamers. The GGTTG sequence, or a similar sequence GGTAG present in the reverse sequence, are predominantly found on the loop structure of aptamers generated against r*Pf*LDH except rLDH 15, demonstrating that this moiety sequence, location and tertiary structure is important in target recognition. The commonality of the GGTTG sequence suggests that aptamers could be designed around this core motif to evaluated the influence of flanking regions. Another sequence, GCGG, was present in 10 of the sequences evaluated here (Table 1). Due to the inherent stability of the GC rich stem-loop, the resulting tertiary structure may play an important role in the specific and high affinity complexation of the aptamers to the *P. falciparum*-specific peptide and recombinant *P. falciparum* LDH. However, further investigation into this particular binding site is required.

Rapid diagnostic tests targeting *Pf*HRP-2 have been reported to not detect some *P. falciparum* parasitaemias and this is due to deletions in the gene from isolates from Peru, India and Africa and due to different amino-acid sequences arising from single nucleotide polymorphisms [37–39]. An analysis of sequences of the *Pf*LDH gene sequence from multiple isolates (personal observation) shows 98.1–100% conservation in the sequence. One sequence had a G87R and the two others a D97L mutation. Both of these changes are outside the peptide targeted in this study. Current sequence evidence shows that the LDH enzyme amino acid structure is highly conserved, as predicted from the essential role the enzyme plays in malaria metabolism [9]. The paucity of amino acid changes in the protein and the location of the two changes identified to date support the potential use of the peptide as a malaria biomarker for diagnosis.

The four aptamers selected to detect rLDH bound well to rLDH with aptamer rLDH 1 detecting both r*Pf*LDH and r*Pv*LDH. Aptamers rLDH 4 and 15, in ELONA, detected only the recombinant r*Pf*LDH protein (Fig. 3). Aptamer rLDH 4 exhibited the highest signal and did not detect mammalian or human LDH or human serum albumin. Both rLDH 4 and 7 detected the native protein in confocal microscopy (Fig. 6a). However, the high absorbance value for HSA by rLDH 7 precludes this aptamer from further investigation. Four out of the five LDHp aptamers detected the peptide and of those, only one, LDHp 11 was able to detect the recombinant protein (Fig. 4) with significant K_D_ values (Table 2). LDHp 11 sensitively detected the native protein in the immunoflourescence study over LDHp 1, rLDH 4, the previously published aptamer, pL1 and control aptamer, C7 (Fig. 6b). None of the five aptamers against the LDHp peptide detected the recombinant *P. vivax* LDH (Fig. 4) while only LDHp 11 showed binding to the recombinant protein. The LDHp 11 aptamer did not detect LDH from mammalian, human or *P. vivax* and no apparent affinity constant could be modelled, supporting the specificity of the aptamer and the selectivity of the target peptide. This data compliments the results obtained with antibodies raised against the same peptide [12]. This species-specificity and selectivity of the aptamer is very promising for the development of a biosensor to detect *P. falciparum* and is an important finding in this study.

The calculated K_D_ value of pL1 to r*Pv*LDH using ELONA is higher than those stated by Lee et al. [20] using fluorescence, and those produced by Cheung et al. [24]. While recent studies [24] determined K_D_ values for pL1 using ELONA (as in the studies herein), differences in experimental conditions such as buffer formulation and pH of the aptamer-protein binding buffer influence the binding kinetics. Both prior studies examining pL1 binding kinetics show a higher affinity of pL1 to r*Pv*LDH, similar to findings herein (Table 2). A key finding of studies presented herein show that an aptamer generated against a species-specific epitope of *P. falciparum* (LDHp 11) demonstrates greater specific binding to the recombinant protein than aptamers generated against the whole recombinant protein.

The data obtained with confocal microscopy (Fig. 6) showed similar distribution of LDH detected by aptamers and IgY, and this corresponded to the images detected with IgY previously [12]. Studies on the distribution of LDH within the parasite probed with IgY and aptamers using immunoelectron microscopy at different stages of parasite intra-erythrocytic development are ongoing. Current research includes screening for *P. vivax* LDH specific aptamers.

When polyclonal antibodies are raised against a whole protein and affinity purified, the pool of antibodies contains multiple antibodies against a range of epitopes on a protein. There are likely to be higher titres of antibodies against one epitope compared to another. Aptamers, on the other hand have an inherent limited region of the target protein. This was illustrated by four aptamers detecting r*Pf*LDH and only one aptamer detecting r*Pv*LDH and none of the aptamers detecting the r*Pf*LDH peptide.

The *P. falciparum* LDH specific peptide was shown with peptide antibodies, to be a suitable antibody target whereby antibodies against the peptide differentiated between a *P. falciparum* and *P. vivax* LDH and by inference between the two species [12]. The peptide based approach has enabled the identification of a *P. vivax* specific LDH peptide and the selection of plasmodial glyceraldehyde-3-phosphate dehydrogenase specific peptides and antibody partners with diagnostic potential [13]. Peptide antibody based diagnostic tests detecting both plasmodial LDH and HRP-2 have been successfully evaluated with field isolates [14, 40]. The use of peptide/antibody combinations for the development of malaria diagnostic tests has been extended in this study to include peptide/aptamer combinations. Aptamers have several advantages over antibody based techniques which can be explored to establish rapid diagnostic tests that are less sensitive to storage temperatures and conditions and are cheap to synthesize.

## Conclusions

Binding properties of selected aptamers to r*Pf*LDH were investigated using ELONA, while in situ binding in *P. falciparum* parasites was demonstrated using fluorescently labelled aptamers and confocal microscopy. ELONA strongly suggested that one aptamer, LDHp 11, differentiated between *Plasmodium* LDH from different species, showing a clear preference for r*Pf*LDH. This is supported by K_D_ values of 321.2 ± 82.5 nM for LDHp 11, to r*Pf*LDH. The application of this particular aptamer as the biorecognition element in biosensors and other diagnostic devices is very promising. Data presented herein concur with Lee et al. [20, 22] and Tanner et al. [21, 23, 24], but extend their findings with the addition of aptamers that differentiate between *P. falciparum* and *P. vivax* LDH and species of malaria employing a species-specific epitope. This is the first aptamer set where the aptamers were selected against a conserved peptide epitope on *P. falciparum* lactate dehydrogenase and the aptamers have specificity to both the larger recombinant LDH protein and the native protein. Of specific import to future studies is that aptamers generated against the species-specific epitope of *Pf*LDH (peptide aptamers) detected only *Pf*LDH and not *Pv*LDH. This study paves the way to explore aptamer generation against targeted species-specific epitopes of other *Plasmodium* species. Future research will explore the use of these generated aptamers in a biosensor assembly using clinical samples.
